# Bacterial co-infections and superinfections in COVID-19: a case report of right heart infective endocarditis and literature review

**DOI:** 10.11604/pamj.supp.2020.35.2.23577

**Published:** 2020-05-20

**Authors:** Rime Benmalek, Hanane Mechal, Hamza Choukrallah, Anas Maaroufi, El Ghali Benouna, Rachida Habbal, Ouissal Aissaoui, Anass Erragh, Afak Nssiri, Rachid AlHarrar

**Affiliations:** 1 COVID-19 Dedicated Cardiology Team, University Hospital Center of Casablanca, Morocco; 2 COVID-19 Dedicated ICU team, University Hospital Center of Casablanca, Morocco

**Keywords:** COVID-19, co-infections, infective endocarditis, multidisciplinary management

## Abstract

Coronavirus disease of 2019 (COVID-19) is a worldwide pandemic with significant morbidity and mortality. Patients with severe forms of the disease are usually managed in the Intensive Care Unit (ICU), where they can develop secondary infections particularly bacterial, favored by prolonged intubation and central venous catheterization (CVC), hence increasing the disease’s mortality. Infectious endocarditis (IE) represents a rare and severe cardiovascular complication in patients with CVC. We report the case of a patient admitted to the ICU for an acute respiratory distress syndrome (ARDS) due to COVID19. Her management included intubation and mechanical ventilation, CVC and treatment with Hydroxychloroquine and azithromycin, and echocardiography findings were unremarkable. On the 10th day of onset, the patient developed septic shock and both echocardiography and blood cultures were in favor of A positive diagnosis of tricuspid valve infective endocarditis, accordingly to the modified Duke criteria. Specific treatment was started with a good clinical evolution. Our case outlines the difficulty of management of bacterial co-infections and superinfections in COVID-19 ICU patients, and particularly rare infections such as right-heart IE, which usually require a multidisciplinary approach and coordination between intensivits, cardiologists and infectiologists.

## Introduction

Coronavirus disease of 2019 (COVID-19), caused by infection from severe acute respiratory syndrome coronavirus 2 (SARS-CoV-2) has officially been declared a worldwide pandemic by the World Health organization in March 2020, with more than 3 millions documented cases in the world and almost 5000 cases in Morocco as of May 1st, 2020 [1] causing significant morbidity and mortality [2]. This disease can cause severe complications, which in addition to its high transmissibility have led COVID-19 to become a serious public-health threat all over the world. While much of the focus has been on the pulmonary complications, particularly the acute respiratory distress syndrome (ARDS), it is important to outline the gravity of cardiovascular complications that are among the most significant and life-threatening [3]. Moreover, secondary infections particularly bacterial are common especially in hospitalized, critically ill COVID-19 patients in the Intensive Care Unit (ICU) favored by prolonged intubation and central venous lines placement, hence increasing the disease´s mortality [4,5]. Infectious endocarditis represents a rare and severe cardiovascular complication in patients with central venous lines. In this case report, we describe the first case of COVID-19 patient complicated with right heart infective endocarditis encompassing both topics of bacterial co-infections and cardiovascular complications of COVID 19 and outlining the importance of multidisciplinary management.

## Observation

**Figure 1:**
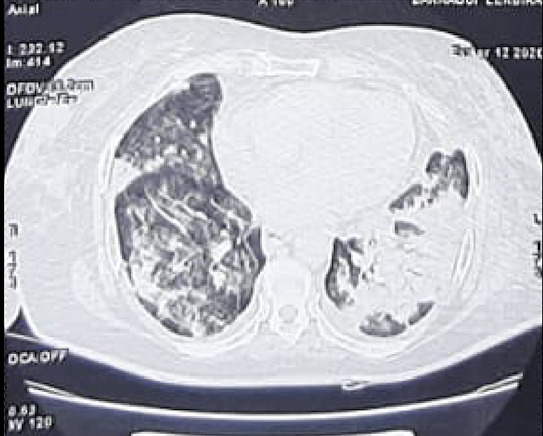
Thoracic CT scan showing bilateral extended alveolar interstitial infiltrates exceeding 75% in the left side in favor of COVID-19

We report the case of a 76-year-old woman, BMI= 30, with hypertension and diabetes mellitus who presented to the emergency department with abdominal pain and shortness of breath, she also reported fever and non-productive cough 5 days before. Physical examination at admission in the ICU revealed blood pressure of 160/90 mmHg, heart rate of 110 beats per minute, oxygen saturation of 89% while breathing ambient air, and body temperature of 36.8°C, capillary blood glucose was superior to 5g/L, and dipstick showed high levels of ketones. The arterial gas analysis showed pH 7.31, PCO2 39.4 mmHg, PO2 68 mmHg, HCO3= 18 mmol/L. The patient´s diabetic ketoacidosis was resolved after insulinotherapy and symptomatic treatment and the evolution was the apparition of severe hypoxia and ARDS requiring orotracheal intubation with mechanical ventilation (Volume assist-control ventilation 6 mL/kg, PEEP= 10 mmHG, FiO2= 80%). Based on the patients´ clinical evolution and the COVID-19 outbreak, a viral pneumonia of SARS-COV-2 was deemed as likely. The thoracic CT scan showed bilateral extended alveolar interstitial infiltrates exceeding 75% in the left lung (Figure 1), PCR of nasopharyngeal swab was performed with a positive result for SARS-CoV-2. Blood tests were in favor of COVID-19 with Lymphopenia and Hyperferritinemia, elevated C-reactive protein and normal procalcitonin. High sensitive troponin I and Brain natriuretic peptide (BNP) levels were normal.

The findings on the 12-lead electrocardiogram (ECG) were unremarkable and transthoracic echocardiography (TTE) showed an aspect of hypertensive cardiomyopathy with an estimated LV ejection fraction (LVEF) of 55%. LV diastolic function was mildly impaired. Right ventricular dimensions and function were normal. We noted a small pericardial effusion most notable around the right atrium (maximum 7mm). Thus, a therapy according to the Moroccan treatment protocol with Hydroxychloroquine and azithromycin was initiated. On the 10th day of onset, the patient presented fever with a temperature of 39.4°C (103°F), and hypotension (84/46 mmHg) requiring vasoactive support (norepinephrin). Blood sample tests showed higher level or High C-reactive protein (432 mg/L) and an important increase in procalcitonin (2,32 ng/mL). Both blood cultures and cultures performed in the patient´s jugular catheter were positive for Coagulase-negative staphylococcus. Cytobacteriological examination of urine showed presence of Candida tropicalis. The patients also presented a biological disseminated intravascular coagulation.

**Figure 2:**
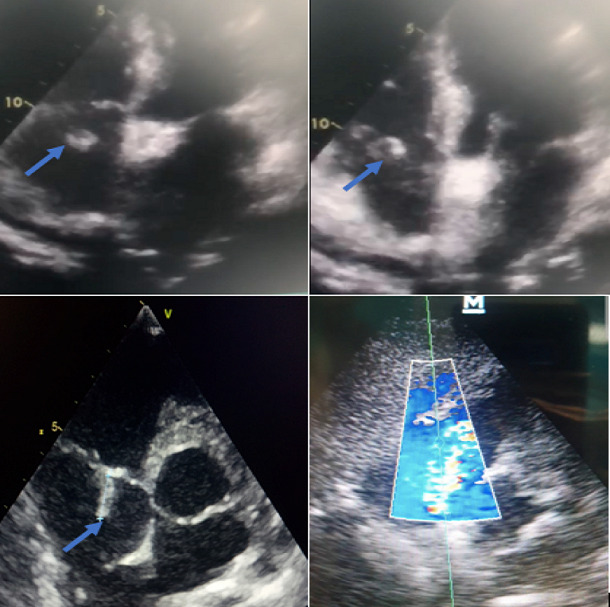
Transthoracic echocardiography images showing oscillating vegetation attached to the anterior leaflet chordate of the tricuspid valve (blue arrow) with a moderate tricuspid regurgitation

**Figure 3:**
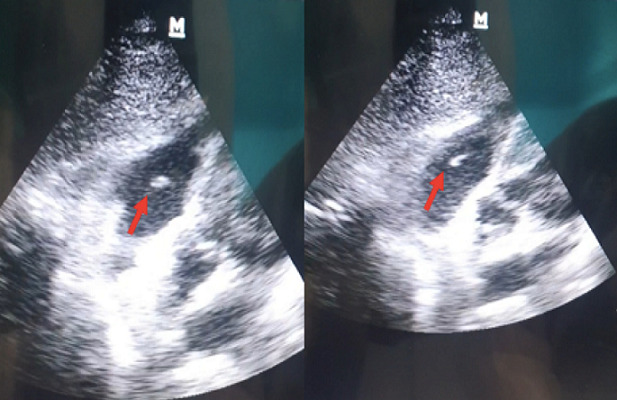
Control transthoracic echocardiography performed 2 weeks after treatment initiation showing a reduction of the tricuspid vegetation size (red arrow)

The TTE was performed (Figure 2) and this time revealed in addition to the first findings a large, oscillating vegetation measuring 15 × 10mm attached to the anterior tricuspid valve leaflet chordate associated with a moderate tricuspid with an estimated pulmonary artery pressure of 35 mmHg. However, no right chambers´ dilatation or right ventricular dysfunction were noted. The diagnosis of tricuspid valve infective endocarditis was thus established accordingly to the modified Duke criteria [6] and antibiotherapy with vancomicyn 30 mg/Kg/day and rifampicin 900mg/day was started. We also added voriconazol 200mg/day for the candida tropicalis. The evolution was good with progressive apyrexia and stabilization of blood pressure, norepinephrin was weaned on the 5th day. On the 21st day, tracheostomy was performed and mechanical ventilation weaning was carried out successfully using a daily T-tube. Control TEE revealed a reduction of the vegetation size (Figure 3), and therefore, surgery was not considered. At the time of submission, the patient was hospitalized with progressive clinical and hemodynamic improvement.

## Discussion

The massive pandemic caused by SARS-COV-2 virus has come with major adjustments in healthcare systems [4]. While secondary infections are well-known in influenza, SARS, MERS, and other respiratory viral diseases, data regarding bacterial co-infections in COVID-19 are still limited. According to recent studies, secondary infections are described as common in hospitalized, severely ill COVID-19 patients with higher frequency in the ICU setting, with a higher incidence of bacterial/fungal secondary infections than viral especially in patients with prolonged intubation where multidrug-resistant Gram-negative bacteria likely reflect nosocomial infection [5]. Moreover, some studies showed that secondary bacterial infections are an important risk factor for adverse COVID-19 outcomes [7]. Zhou et al study found that 50% of COVID-19 patients who died had secondary bacterial infections and all the patients received empirical antibiotic treatment [4]. Ruan et al study analyzed predictors of mortality among 150 patients in Wuhan, assessing that among 68 deaths, 11 patients (16%) had secondary infections [8].

Patients may also be suffering from secondary co-infections, not linked to their respiratory presentation, for example urinary tract or blood stream infection and even of their heart components. In our case, the patient presented septic shock on the 10th day of onset, and TEE revealed a tricuspid valve vegetation with the presence Coagulase-negative staphylococcus in both blood and jugular central catheter cultures. Infective endocarditis (IE) is defined by microbial infection of the heart valves (native or prosthetic), the endocardial surface or intra-cardiac devices [6]. IE incidence has not significantly changed over the past 30 years [9] and still remains an important clinical issue with an important mortality rate (up to 40%). Although rheumatic heart disease represents an important predisposing factor for IE in developing countries such as Morocco, degenerative valve disease, valves´ surgery and nosocomial infection are the major changes in IE incidence [10]. Usually, native IE affects the left heart. Isolated native right-sided IE (RSIE) referring to IE involving the tricuspid or pulmonic valve, account for approximately 10% of all IE cases [10,11] and the tricuspid valve is involved in more than 90% of RSIE cases [11]. Tricuspid valve IE and RSIE in general is a disease predominantly found in intravenous drug abusers although medical device implantation, including pacemakers or defibrillators, and the presence of an intravascular device such as central venous catheterization (CVC) or ventricular assist device, are also major risk factors. Patients with congenital heart disease or underlying right-sided cardiac anomaly are also at increased risk for RSIE [6].

In our situation, the risk factor for RSIE was most likely the jugular CVC. In fact, patients with cardiac implantable electronic device (CIED) or intravascular device such as CVC account for approximately 9% of patients with RSIE depending on the type of catheter, frequency of catheter manipulation and patient-related factors [12]. In this case, RSIE can be secondary to bacterial contamination at the time of implantation or subsequent handling. Moreover, CVC are more likely to cause IE when the tip of the catheter is deep in the right atrium. The potential mechanism may be the abrasion of the tricuspid leaflet and endocardium due to the proximity of fully inserted CVC to the tricuspid valve, and forceful and rapid jet of injections through the catheter [13]. In our patient´s case, the CVC inserted deep in the right atrium, in addition to the direct irritation of the tricuspid leaflet, our patient immunodepression and the hypercoagulability state linked to COVID-19, may all have participated in causing IE in our patient. Concerning the microbiology, RSIE is attributable to Staphylococcus aureus in more than 70% of cases. Streptococci and enterococci are incriminated in 5 to 30% and 2 to 5% of cases respectively [6]. However, coagulase-negative staphylococci represent 25% of CIED-related infections [6,10,14].

Clinical manifestations caused by RSIE are usually inconstant and can be missed easily. The diagnosis is suspected in patients with risk factor like intravascular device who present with fever, with or without respiratory symptoms. The confirmation of the diagnosis is based on clinical manifestations, blood cultures and echocardiographic findings [6]. A definitive diagnosis of RSIE is established when the patient has positive blood cultures with echocardiographic evidence of right-sided vegetation. RSIE diagnosis may rely on the modified Duke criteria [6]; however, these criteria, which were primarily developed for left-sided IE, may be difficult to assess in RSIE and have low sensitivity in patients with suspected RSIE and cardiac device infection [15]. Moreover, the right heart has many anomalous anatomic features that may be confused with vegetations in the TEE. Concerning the treatment, the decision to start antibiotherapy must be individualized and discussed with the Endocarditis team while waiting blood cultures results. In Hemodynamically unstable patients with clinical presentation in favor of IE, empirical antibiotic therapy can be initiated (Grade 2C) [6]. For patients who are clinically stable, antimicrobial therapy may be adjourned while awaiting blood culture results and can be adjusted according to the antibiotic sensitivity spectrum results. In our case, the blood cultures showed a multiresistant S. Coagulase negative sensitive to vancomycine and rifampicine, this bitherapy was thus initiated. Finally, RSIE prognosis is relatively good remains better than left-sided IE. 70-85% of patients have a cleared bacteremia under medical treatment alone. Between 5-16% of RSIE cases eventually require surgery, with reported operative mortality between 0-15% for patients with isolated tricuspid valve IE [6]. In our case, the patient responded well to medical treatment, therefore, surgery was not considered.

## Conclusion

In the present COVID-19 pandemic context, the severely-ill patients with ARDS hospitalized in the ICU are often predisposed to secondary infections particularily bacterial due to several risk factors such as intubation or central veinous lines. Tricuspid valve IE is a rare and severe cardiovascular complication in patients with CVC. Moreover, immunodepression and pro-thrombotic state associated with COVID-19 may be associated risk factors for IE. The management of the association COVID-19 and IE in the ICU represents a true challenge requiring a multidisciplinary approach and a close coordination between intensivists, cardiologists, infectiologists and radiologists.

## Competing interests

The author declares no competing interests.
